# Editorial: Facing the Other: Novel Theories and Methods in Face Perception Research

**DOI:** 10.3389/fnhum.2016.00032

**Published:** 2016-02-08

**Authors:** Davide Rivolta, Aina Puce, Mark A. Williams

**Affiliations:** ^1^School of Psychology, University of East LondonLondon, UK; ^2^Department of Psychological and Brain Sciences, Indiana UniversityBloomington, IN, USA; ^3^Perception in Action Research Centre, and ARC Centre of Excellence in Cognition and its Disorders, Department of Cognitive Science, Faculty of Human Sciences, Macquarie UniversitySydney, NSW, Australia

**Keywords:** face recognition, holistic processing, Prosopagnosia, fMRI methods, EEG/MEG, fNIRS, Philosophy, MVPA

Human and non-human primates rely on information gathered from faces during social interaction. Two channels of information are gathered from the face—the *identity* of the individual (conveyed by featural and configural aspects of the face), as well as their *mental state and potential intentions* (conveyed by the dynamic face). With respect to identity, humans can recognize thousands of people who are familiar to them very quickly and accurately without effort. Similarly, new individuals can be identified often after only one previous encounter. With respect to the affective/mental states of others, these can be inferred from affective expressions as well as other facial signals such as gaze changes from both strangers and those who are well known to us.

That brain injuries can cause selective deficits in the recognition of identity was at the core of a now classic model of face perception proposed by Vicki Bruce and Andy Young in the late 1980s (Bruce and Young, 1986). The original model also postulated a pathway for dealing with facial expressions. Indeed, further work on the affective aspects of face processing was conducted by Andy Calder in collaboration with Andy Young (Calder and Young, 2005). The elements of this model have been given a parallel processing and functional neuroanatomical bent, based on not only patient studies, but on functional neuroimaging investigations showing ventral visual pathway activity in healthy subjects (Haxby et al., 2000). Multivariate classification methods (e.g., MVPA) analyzing fMRI data further indicate that category-specific patches of cortex, such as those observed to faces, may be identified in the ventral visual system (Haxby et al., 2001), consistent with human intracranial neurophysiological studies (Puce et al., 1999).

In the current “Research Topic” we have gathered 33 works that include experimental studies, as well as hypothetical and theoretical contributions that review the various behavioral, neurophysiological, and hemodynamic correlates of face processing across the lifespan in both typical and atypical populations. It is our view that future important achievements in the field will likely be derived via interdisciplinary collaborations of scientists coming from different fields, as evidenced by the manuscripts in this volume that include contributions from social and cognitive neuroscientists, cognitive scientists, clinical psychologists, philosophers, as well as engineers, and physicists. Overall, face processing in healthy subjects formed the major corpus of manuscripts in the current “Research Topic.” Prosopagnosia was the main theme of six research studies (see below) and two reviews on strategies to enhance face-processing skills (Bate and Bennetts; DeGutis et al.), thus representing the most investigated condition in this special issue on face processing.

Clinical conditions such as social anxiety, epilepsy, attention deficit hyperactivity disorder (ADHD) and autism have also received attention in this “Research Topic”. In individuals with social anxiety, skin conductance recordings show enhanced unconscious threat processing relative to those from neurotypical individuals (Jusyte and Schonenberg). Furthermore, emotion processing has been discussed in light of threat detection (Holt et al.) and in relation to the genesis and maintenance of psychopathology (Tanzer et al.). Finally, individuals with ADHD and autism have been differentiated using a novel classification method of hemodynamic responses (as measured with functional near infra-red spectroscopy; fNIRS; Ichikawa et al.).

Face perception, particularly with respect to gleaning an individual's identity, has long been proposed to engage a holistic/configural type of processing, which involves the analysis of the face as a whole, rather than processing individual features in isolation (Young et al., 1987; Maurer et al., 2002; McKone and Yovel, 2009). Holistic/configural processing has been deduced, amongst others, from studies of the “face inversion effect” (i.e., greater difficulty perceiving facial identity in inverted relative to upright faces; Yin, 1969) and the “composite face effect” (i.e., reduced facial identification performance when face halves are vertically aligned compared to when they are misaligned; Young et al., 1987). Profound facial identification deficits (i.e., prosopagnosia) are known to follow injuries to part of the ventral occipito-temporal cortex, seriously undermining the ability of the affected individuals to maintain normal social interactions (Barton, 2008; Rossion, 2008). A more puzzling problem is that of facial recognition deficits that have been present from birth in individuals with no known neurological disease—a condition known as congenital or developmental prosopagnosia (CP; Duchaine, 2000; Behrmann and Avidan, 2005; Rivolta et al., 2013). Face identity recognition can also be more difficult (i.e., increased response time, reduced performance) when the face belongs to an individual from a different race from a neurotypical healthy subject; this is known as the “Other Race Effect” (ORE; Meissner and Brigham, 2001).

In the current “Research Topic”, the importance of holistic/configural processing for typical face perception has been underlined in two contributions showing its impairment in both CP (Liu and Behrmann) and in acquired prosopagnosia (AP; Jansari et al.). Using tasks that tapped into holistic/configural processing, such as the composite faces task (Liu and Behrmann), the Navon task and the face-fracturing test (Jansari et al.), these studies demonstrate similar deficits in face processing in a group of individuals who have had face processing deficits since birth (i.e., CP) and in an individual who acquired his deficit much later in life (i.e., AP). In a contribution investigating the ORE, Esins et al. show that despite its apparent similarity to CP, the ORE and CP are unlikely to share the same cognitive mechanism.

Additionally, five studies in the current issue have attempted to further delineate characteristics of holistic/configural face processing in healthy subjects in terms of the experimental design of the composite face task (Meinhardt et al.), of the physical properties of the face itself (Persike et al.; Stein et al.), and of the familiarity of the face (Liccione et al.; Visconti di Oleggio Castello et al.). In sum, results demonstrate: (1) the validity of the complete-design of the composite face task (Meinhardt et al.); (2) a stronger inversion effect for faces than for houses when assessed using opponent-stimulus rivalry (Persike et al.); (3) stronger face-detection mechanisms for same-race and same-age faces when assessed by continuous flash suppression (Stein et al.); (4) a critical role of face familiarity (especially for personally familiar people such as family members) in driving a stronger face-inversion effect (Liccione et al.) and in driving better detection of social cues (Visconti di Oleggio Castello et al.). Finally, novel philosophical accounts based on the phenomenological tradition (e.g., Heidegger, 1996; Marleau-Ponty, 2002) and on work from Levinas (e.g., Levinas, 1969) that mainly focus on the embodied nature of humans have been proposed to re-interpret studies of typical and atypical face processing (Gallagher; Liccione et al.).

Additionally, two novel investigations of face processing with methods such as hypnosis (Connors et al.), and adopting an individual difference approach, when dealing with very large samples (Huang et al.; Yovel et al.) round out the studies dealing with the holistic/configural processing of faces. These studies highlight that important information can be gleaned from between-subject variance in datasets. The evidence that face recognition ability varies across individuals and dissociates from other cognitive abilities is explored as a model that may result in the discovery other specific abilities (Wilmer et al.).

Given the distributed system for processing face identity and expression in the human brain (Haxby et al., 2000), the current “Research Topic” also features a series of contributions that investigate interactions between face identity, face expression, and body expression in neurotypical subjects (Van den Stock and de Gelder; Vicario and Newman; Yankouskaya et al.), and in individuals with CP (Daini et al.). Results in control participants demonstrated that: (1) task-irrelevant bodily expressions influence face-identity matching performance (Van den Stock and de Gelder); (2) emotional-face primes affect the perception of emotional hand gestures (Vicario and Newman); (3) face identity and expression interact when assessed with the Garner paradigm, the composite face task, and the divided attention tasks (Yankouskaya et al.). In contrast, people with CP were impaired in detecting the identity of unfamiliar faces, but not in the detection of non-emotional facial expressions, thus suggesting a dissociation between changeable and invariant configural processing in CP (Daini et al.). Additionally, Kim et al. show that that MVPA is more sensitive than traditional univariate analysis for characterizing the spatial distribution of face- and body-specific activations in the human brain. These results have been corroborated in a second paper (Rivolta et al.) that additionally demonstrated aberrant face versus object activation patterns in CP compared to typical face recognizers. Intracranial EEG recordings in drug-resistant epileptic patients posit that eye-sensitive brain regions are actually more abundant and more selective than brain regions that are face- and body- sensitive (Engell and McCarthy).

Neurophysiological studies over twenty years ago demonstrated that a specific negative potential at around 170 ms post-stimulus onset can index aspects of face processing. This ERP was first demonstrated in scalp EEG recordings by Shlomo Bentin and his colleagues, and is known as N170 (Bentin et al., 1996), and in intracranial EEG (N200) by Truett Allison and his team (Allison et al., 1994). The magnetic analog of N170 can also be recorded with magnetoencephalography (MEG), and this entity is known as M170 (Liu et al., 2002; Rivolta et al.). N/M170 is not the only component to show face-sensitive properties—other ERP components have also been described in adults and also in older children (Taylor et al., 2004; Rivolta et al., 2012, 2014; Rossion, 2014). The later components involved in recollection and familiarity of faces, were also explored in CPs, demonstrating abnormal neural processing during face recognition in these individuals compared to controls (Burns et al.).

How can the neurophysiological data inform our understanding of face processing in the human brain? Hemodynamic studies have identified the neuroanatomical substrates for face processing in the human brain. MEG and EEG studies have the capability to characterize the *timing* underlying these processes (Buzsáki et al., 2012). In the current “Research Topic,” N/M170, and other face components, were studied during holistic/configural processing (Marinkovic et al.; Vakli et al.). Reduced gender-adaptation from stretched faces (a manipulation that affects holistic/configural processing) as compared to normal faces (Vakli et al.), and increased and delayed M170 in the right posterior fusiform gyrus (Marinkovic et al.) for inverted faces was found (in line with earlier scalp EEG studies, e.g., Bentin et al., 1996). N170 recordings to eyes and upright and inverted faces in Japanese children indicate that an adult neurophysiological pattern is not seen in children that are younger than 13 years of age (Miki et al.). Interestingly, Nakabayashi and Liu have re-examined the developmental behavioral literature and make the claim that holistic processing is present in early childhood, indicating that some future studies will need to reconcile behavioral and neurophysiological data.

Social context influences how neurophysiological activity to emotional expressions manifests. Specifically, as early as N170, augmentation of the neural response occurs to non-neutral expressions in faces that have been designated as future partners for a social interaction. These data clearly indicate how top-down processing can modulate sensory activity (Bublatzky et al.).

As already noted, neurophysiological methods can identify the timing of neural activity and its dynamics. Given that this is the case, these methods are ideal for studying activity elicited to dynamic faces. Rossi and colleagues show that augmented N170s to viewed dynamic gaze aversions occur to real but not impoverished faces, suggesting that local scleral/iris luminance and contrast plays a role in generating these responses. Additionally, bursts of gamma activity at around 200 and 300 ms post-motion onset may signal detection of facial motion (Rossi et al.). There is a need for more studies evaluating both the dynamics of the MEG and EEG signals and ERP measures so that the earlier and more recent literatures can be bridged. In a similar fashion, comparing data in the same subjects viewing static and dynamic faces (the former in highly controlled lab setting and the latter in more ecologically valid contexts) is greatly needed.

The current “Research Topic” evolved over the desire to acknowledge the relatively recent loss of three giants in the field: Drs. Shlomo Bentin, Truett Allison, and Andy Calder. Shlomo Bentin was fascinated by the holistic/configural aspect of face processing, Andy Calder was stimulated to study how the brain deals with affective facial information, and Truett Allison was interested in the functional neuroanatomy of both facial processing streams—identity and affect. All three scientists were known for working with multiple assessment methods and varied subject populations. We dedicate this “Research Topic” to them and their pioneering studies.

## Author contributions

All authors listed, have made substantial, direct and intellectual contribution to the work, and approved it for publication.

### Conflict of interest statement

The authors declare that the research was conducted in the absence of any commercial or financial relationships that could be construed as a potential conflict of interest.
